# Hierarchical Nanoheterostructure of HFIP-Grafted α-Fe_2_O_3_@Multiwall Carbon Nanotubes as High-Performance Chemiresistive Sensors for Nerve Agents

**DOI:** 10.3390/nano14030305

**Published:** 2024-02-02

**Authors:** Xuechun Wang, Jingyuan Liu, Rumin Li, Jing Yu, Qi Liu, Jiahui Zhu, Peili Liu

**Affiliations:** Key Laboratory of Superlight Material and Surface Technology, College of Materials Science and Chemical Engineering, Harbin Engineering University, Harbin 150001, China; wxcup0202@163.com (X.W.); lirumin@hrbeu.edu.cn (R.L.); jing.yu@hrbeu.edu.cn (J.Y.); qiliu@hrbeu.edu.cn (Q.L.); jianhuizhu@hrbeu.edu.cn (J.Z.)

**Keywords:** chemical warfare agent, DMMP gas sensor, multiwall carbon nanotubes, α-Fe_2_O_3_ nanorods, nanoheterostructure

## Abstract

New and efficient sensors of nerve agents are urgently demanded to prevent them from causing mass casualties in war or terrorist attacks. So, in this work, a novel hierarchical nanoheterostructure was synthesized via the direct growth of α-Fe_2_O_3_ nanorods onto multiwall carbon nanotube (MWCNT) backbones. Then, the composites were functionalized with hexafluoroisopropanol (HFIP) and successfully applied to detect dimethyl methylphosphonate (DMMP)-sarin simulant gas. The observations show that the HFIP-α-Fe_2_O_3_@MWCNT hybrids exhibit outstanding DMMP-sensing performance, including low operating temperature (220 °C), high response (6.0 to 0.1 ppm DMMP), short response/recovery time (8.7 s/11.9 s), as well as low detection limit (63.92 ppb). The analysis of the sensing mechanism demonstrates that the perfect sensing performance is mainly due to the synergistic effect of the chemical interaction of DMMP with the heterostructure and the physical adsorption of DMMP by hydrogen bonds with HFIP that are grafted on the α-Fe_2_O_3_@MWCNTs composite. The huge specific surface area of HFIP-α-Fe_2_O_3_@MWCNTs composite is also one of the reasons for this enhanced performance. This work not only offers a promising and effective method for synthesizing sensitive materials for high-performance gas sensors but also provides insight into the sensing mechanism of DMMP.

## 1. Introduction

In the tranquil English city of Salisbury, Sergei Skripal, a former Russian military officer, and his daughter Yulia, fell victim to poisoning in March 2018, attributed to the use of a nerve agent called Novichok. The incident reawakened alarm and concern about nerve agents. Nerve agents were developed on the basis of the rapid development of organophosphorus chemistry and organophosphorus insecticides in the 1930s [1,2]. Such agents can strongly inhibit the activity of acetylcholine, which is an important chemical medium in the nervous system. Nerve agent molecules cannot be hydrolyzed by enzymes, leading to the destruction of the normal dynamic process of acetylcholine release and hydrolysis, as well as the normal nerve impulse transmission process [3,4]. Excessive accumulation of acetylcholine in the body leads to a series of toxic reactions throughout the nervous system, which is extremely harmful [5]. Unfortunately, due to their strong toxicity, fast action, easy production, good performance, and its ability to be absorbed through the skin, mucous membranes, gastrointestinal tract and lungs, and other ways to cause systemic poisoning [6], nerve agents have become the main chemical warfare agents used by foreign militaries [7]. Despite a convention on the prohibition of the use of chemical weapons, there are still terrorists who use them to launch attacks against civilians, seriously endangering human security and world peace [8]. Sarin, a typical organophosphorus nerve agent, suffocates to death within 1–10 min at exposure concentrations of more than 60 ppb (parts per billion) [3,9]. Because these nerve agents are colorless, odorless, volatile, and act quickly, and human senses are unable to recognize them [10]. Therefore, it is crucial to find a fast and accurate electronic nose that is able to distinguish and sense nerve agents efficiently [11]. To ensure that the experiment is safe, dimethyl methylphosphonate (DMMP), which is similar to sarin in molecular structure but has little toxicity, was selected as a simulation gas for sarin. The chemical formula of sarin and DMMP are C_4_H_10_FO_2_P and C_3_H_9_O_3_P, both belonging to the organophosphorus compound family [12,13,14,15]. -P=O- is an important detection group in the chemical structure of DMMP, and further research on this analog is helpful to provide a reference and basis for the detection of real nerve agents such as sarin [15].

In various fields, carbon materials like carbon nanotubes (CNTs), graphene and carbon fiber have gained extensive usage in recent years due to their excellent physical, chemical, electronic, and mechanical properties in addition to environmental friendliness and lightweight [16,17,18]. Carbon-based sensing materials have also been used by researchers in the field of gas sensors [19]. According to the number of tube wall layers, CNTs can be divided into single-walled carbon nanotubes, double-walled carbon nanotubes, and multi-walled carbon nanotubes (MWCNTs). CNTs are considered an outstanding gas-sensitive material because of their large specific surface area, large number of chemical reaction places, high carrier mobility, and good chemical stability. They have great prospects in the development of chemical and biological sensors [20,21,22]. CNTs are p-type semiconductor materials in which electrical properties change when chemical molecules are adsorbed to their surface [23]. This behavior is based on the operating principle of semiconductor resistive gas sensors [24]. However, bare CNT-based gas sensors have some restrictions, such as limited sensitivity, the absence of selectivity, and a prolonged recovery time [25,26], which limit their practical application. After further investigation, it was found that the sensing performance of CNTs can be seriously affected by surface defects and residual contaminants [27]. Therefore, oxidizing CNTs and grafting specific chemical groups onto CNTs have been considered beneficial strategies to modify their chemical properties and improve their sensing properties [28].

Metal oxide semiconductor (MOS) gas sensors have been widely researched due to their outstanding sensing properties and simple manufacturing process [29,30]. The rapid development of nanotechnology provides an opportunity to realize the powerful aspects of the nanocrystallization of sensitive materials [31]. Although hollow [32], hierarchical [33], core–shell [34] and other special nanostructured sensitive materials show excellent sensing properties due to their large specific surface area, many numbers of active sites, and excellent permeability [35]. However, working at high temperatures makes it difficult to maintain these structural advantages for a long time, which impacts long-term stability and limits the practical application of the sensor. Recently, it was found that constructing MOS nanocrystals on the surface of CNTs creates a synergistic effect between CNTs and MOS semiconductor properties that enhance their sensing properties [25,28,36]. In addition to that, due to the excellent mechanical strength of CNTs, a heterostructure composed of CNTs and MOS can maintain its structural stability for a long time [37]. In the last few years, the gas-sensing properties of CNTs combined with various MOS as heterostructure composite gas-sensing materials synthesized by different methods have been intensively studied [38]. Yang et al. prepared ZnO nanoparticle-coated SWCNTs network sensors for the detection of DMMP using RF magnetron sputtering. The ZnO-SWCNTs network sensor has excellent DMMP sensing behavior even at room temperature. The response performance of the sensor could be transformed from p-type to n-type based on how long the deposition lasts, except for low-temperature detection [39]. Wang et al. prepared an acetone sensor based on a Co_3_O_4_/MWCNT composite material, and the optimized sensor has an ultra-low detection limit of 0.41 ppm. The formation of a p-p heterojunction in the composite greatly improved the detection performance, resulting in an improvement of five times compared to the original Co_3_O_4_ sensor [40]. A ZnO/CoNiO_2_ hollow nanofiber sensor successfully achieved a high response (240) to 100 ppm of target gas at an optimal temperature of 220 °C [41].

The organic group hexafluoroisopropanol (HFIP) is considered to be a facilitator for detecting DMMP by forming hydrogen bonds (H-bonding) with DMMP’s phosphate esters. Based on our expertise and the literature, we chose to graft it onto the composite material in order to improve the selectivity of DMMP [3,10,42]. K.T. Alali et al. reported high selectivity and excellent performance of the double-layer HFIP-rGO/Co_3_O_4_ sensor for detecting 0.5 ppm DMMP compared to bilayer rGO/Co_3_O_4_ with a response of 11.8 at 125 °C. Moreover, the functionalized double-layer sensor maintained 75% of its initial response even at 85% relative humidity [43].

Therefore, in this paper, the synthesis of α-Fe_2_O_3_ nanorods assembled onto MWCNTs backbones by hydrolysis reaction and annealing is proposed to prepare α-Fe_2_O_3_@MWCNT nanoheterostructure composites. Then, HFIP groups were grafted on the surface of α-Fe_2_O_3_@MWCNTs nanocomposites by chemical treatments for rapid and selective detection of DMMP. The HFIP grafted α-Fe_2_O_3_@MWCNTs nanocomposite outperformed any of its individual units for DMMP sensing. α-Fe_2_O_3_ nanocrystal growth on MWCNTs adds to the number of active places for the adsorption of DMMP molecules versus bare MWCNTs and α-Fe_2_O_3_ nanostructures. Hydrogen bonding interactions between HFIP groups and DMMP further enhance its DMMP selective sensing. Other factors that enhance the sensing performance are the generation of a heterogeneous structure and the cooperative effect between MWCNTs and α-Fe_2_O_3_ nanorods.

## 2. Experimental Section

Details of the full range of chemical reagents and characterization equipment are described in the Supplementary Material.

### 2.1. Fabrication of α-Fe_2_O_3_ and α-Fe_2_O_3_@MWCNT Compounds

**Figure 1:**
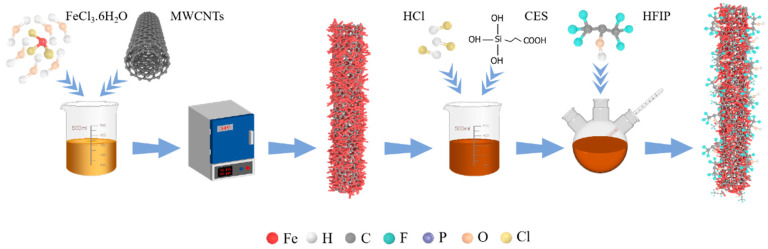
Illustration diagram of the synthesis and HFIP chemical functionalization of prepared HFIP-α-Fe_2_O_3_@MWCNTs.

It is well known that carboxyl (-COOH) and hydroxyl (-OH) groups usually adhere in small amounts on the surface of carbon materials [38]. In order to enhance the number of carboxyl groups on the surface of the MWCNTs, the MWCNTs were subjected to reflux in a mixture of acids. Specifically, 0.5 g of MWCNTs was refluxed in a 20 mL acidic solution comprising 2 mL of HNO_3_ and 6 mL of H_2_SO_4_ for 5 h at a temperature of 40 °C. Later, the resulting samples were flushed alternately with ethanol and deionized (DI) water until the liquid reached a neutral pH and dried overnight. To grow the FeOOH nanospindles on the MWCNTs skeleton, the acid-treated MWCNTs were homogeneously dispersed in an FeCl_3_ solution and underwent a reaction at 80°C for a duration of 5 h. Subsequently, the samples were separated by centrifugation, flushed alternately with DI water and ethanol, and dried overnight at 60 °C. Finally, after annealing in air at 400 °C for 10 h with a slow ramp rate of 2 °C min^−1^, FeOOH was successfully converted to α-Fe_2_O_3_ to obtain the α-Fe_2_O_3_@MWCNTs hierarchical nanoheterostructure. Schematic diagrams of the synthesis process and the final structure are illustrated in Figure 1.

### 2.2. Functionalization with HFIP Chemical Group

The presence of a number of -COOH groups on the surface is a necessary condition for grafting HFIP groups on α-Fe_2_O_3_@MWCNTs [10,38]. To create -COOH groups, 0.5 g α-Fe_2_O_3_@MWCNT nanomaterials were dissolved in 30 mL DI water, the solution’s acidity was lowered to a pH of 4–5 with HCl, and stirred at 40 °C for 30 min [44]. Subsequently, 1.1 mL of carboxyethylsilanetriol was gradually added into the solution and stirred for 1 day at 40 °C. Then, the solution was centrifuged several times with DI water and acetone and dried in a vacuum oven at 45 °C overnight to obtain α-Fe_2_O_3_@MWCNTs–COOH. Subsequently, to graft HFIP groups on the α-Fe_2_O_3_@MWCNTs–COOH, 0.06 g from the as-prepared α-Fe_2_O_3_@MWCNTs-COOH and 0.1 mL of hexafluoroacetone trihydrate were added to 20 mL of N,N-Dimethylformamide (DMF), next the solution was magnetically stirred in a cold-water bath (≈−5 °C) for 3 h. Then, 0.7 g of N-ethyl-N-(3-dimethylaminopropyl) carbodiimide hydrochloride (EDCl), 0.06 g of Hydroxybenzotriazole (HOBt), and 0.6 mL of triethylamine were subsequently added to the former solution and stirred in a cold-water (−5 °C) bath for 3 h and at room temperature (20 °C) for 1 day. In the end, the collected material was centrifuged, washed with DI water, and dried at 45 °C for 1 day. The fabrication and the methodology of HFIP functionalization α-Fe_2_O_3_@MWCNTs are depicted in Figure 1.

### 2.3. Fabrication of Gas Sensors

**Figure 2:**
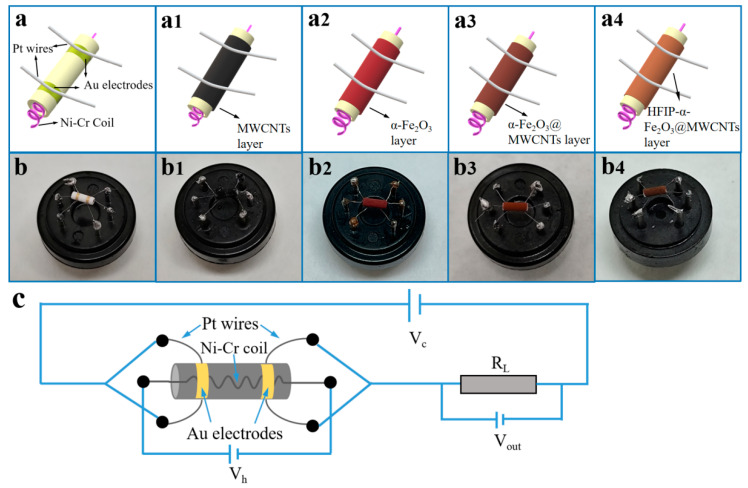
(**a**,**b**) Photos and schematics of the sensors before and after covering with sensing materials, respectively, and (**c**) illustration diagram of the sensor circuit.

To prepare the gas sensors, it was necessary to coat the prepared sensitive material onto the external surface of the ceramic tube, forming a thick sensing film. The sensitive materials need to be covered on the external surface of the ceramic tube to create a dense sensing film [45]. The illustration of the sensor structure in Figure 2 shows the dimensions of the ceramic tube, which are 0.8 mm internal diameter, 1.2 mm external diameter, and 4 mm length. A couple of Au electrodes are fixed at the ends of the tube, and each electrode is attached to a Pt wire. Then, appropriate quantities of MWCNT, α-Fe_2_O_3_, α-Fe_2_O_3_@MWCNTs, and HFIP-α-Fe_2_O_3_@MWCNTs were dispersed in anhydrous ethanol and ground into even pastes. Then, the sensing materials were carefully painted on a ceramic tube to connect the Au electrodes. Subsequently, the sensors were left in the air for 30 min, annealed at 400 °C for 2 h and aged for 7 days before conducting gas measurements. In the electrical circuit of sensors, the resistors of the sensor and load (R_l_) are linked in series, which supplies the circuit voltage (V_c_). The Ni-Cr coil provides the heating voltage (V_h_). Figure 2 displays the electrical circuit schematic of the sensor. When sensors are in contact with the target gas, the chemical adsorption reaction between the sensing material and gas molecules will result in a variation in the resistance of the sensors’ circuit, and this alteration is then detected and presented as an output voltage (V_out_) [46].

## 3. Characterization of Materials and Discussion

**Figure 3:**
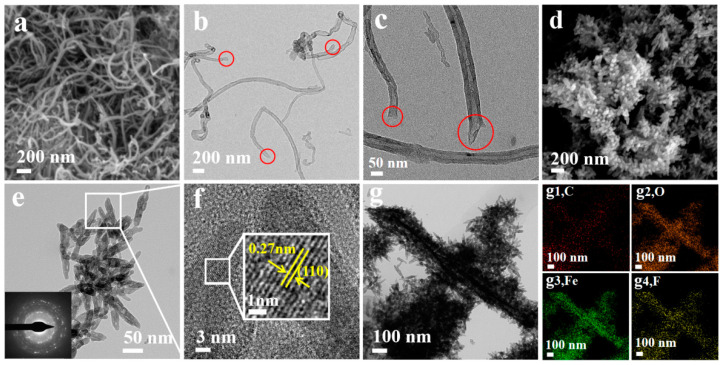
Structural characterization of the prepared samples, (**a**,**b**) SEM and TEM images of MWCNTs; (**c**) TEM image of MWCNTs-COOH; (**d**) SEM images of the α-Fe_2_O_3_@MWCNTs composites; (**e**) TEM image of the α-Fe_2_O_3_@MWCNTs composites and corresponding SAED pattern inset on f; (**f**) HRTEM image of the α-Fe_2_O_3_@MWCNTs composites; (**g1**–**g4**) TEM and TEM mapping patterns of HFIP-α-Fe_2_O_3_@MWCNTs, respectively.

Figure 3a,b show SEM and TEM images of the original MWCNTs, with the regular smooth surface and curled shape. After oxidization by the mixed acids (TEM image is shown in Figure 3c), a slight change in the morphology of MWCNTs could be observed with significant corroding and extension of the ends of the carbon nanotubes. In addition to that, the observation of some micro-folded regions in the MWCNT wall means that MWCNTs have been disrupted to be like multi-layer graphene regions, which highly enhance the sensing performance due to their high functionalization [47]. These changes and the unzipping of MWCNTs are due to the fact that the oxidizing effect of the mixed acid broke the unstable five-membered and seven-membered carbon rings located at the bends of the MWCNTs, shortening and opening the structure of the MWCNTs, effectively increasing the surface area-to-volume ratio. The SEM of the α-Fe_2_O_3_ nanoparticles in Figure 3e shows that they are in a seed-like structure with an approximated size of 30 × 100 nm. The SEM image of α-Fe_2_O_3_ @MWCNTs in Figure 3d shows that the α-Fe_2_O_3_ are completely grown and have warped the skeleton of the MWCNTs, forming layered rod-like structures. However, since MWCNTs are easily wound, these rod-like structures are interleaved and stacked together. The corresponding selective electron diffraction (SAED) (lower left corner of Figure 3e) and HRTEM characterization confirmed that the α-Fe_2_O_3_ nanorods are polycrystalline, and the lattice fringes can be clearly observed in Figure 3f with lattice spacings of 0.27 nm, which is consistent with the (110) crystal face spacing of α-Fe_2_O_3_ crystal, respectively. The TEM image of α-Fe_2_O_3_@MWCNTs after functionalization by HFIP is shown in Figure 3g, and the overall structure of the material remains unchanged after annealing. The successful grafting of HFIP on α-Fe_2_O_3_@MWCNTs was confirmed by the TEM mapping patterns of C, O, Fe, and F elements in Figure 3(g1–g4). [3,10,42]. In the product α-Fe_2_O_3_@MWCNT composite material, it is difficult to directly observe the presence of MWCNTs in the SEM and TEM images due to the fact that MWCNTs are tightly wrapped by α-Fe_2_O_3_ nanorods. The TEM mapping patterns of C also confirm the presence of MWCNTs.

**Figure 4:**
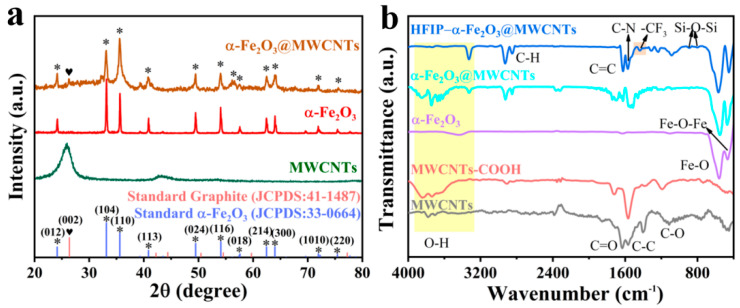
(**a**) XRD patterns of the α-Fe_2_O_3_@MWCNTts nanostructures, α-Fe_2_O_3_ and MWCNTs; (**b**) FT-IR spectra of the representative samples at different preparation stages.

The crystal phase and purity of all samples (MWCNTs, α-Fe_2_O_3_, α-Fe_2_O_3_@MWCNTts) were confirmed by XRD patterns, as shown in Figure 4a. A series of intense and sharp peaks are evident in all diagrams. Except that the diffraction peak of 2θ = 25.96° belongs to the (002) crystal face of MWCNTs, all the other diffraction peaks perfectly match α-Fe_2_O_3_ and are consistent with the standard card (JCPDS: 33-0664). No diffraction peaks belonging to impurities were found, confirming the purity of the product. The peak of the α-Fe_2_O_3_@MWCNT composite at 2θ = 26.3° is consistent with the peak of MWCNTs in the (002) direction, indicating that the hydrated ferric oxide was completely transformed into hexagonally crystalline α-Fe_2_O_3_ after thermal treatment and there was no change in the structure of MWCNTs in the composite. The low intensity of the MWCNT peak is due to their low percentage and being covered with the outer layer of α-Fe_2_O_3_ nanoparticles. The FT-IR spectra of MWCNTs, α-Fe_2_O_3_, α-Fe_2_O_3_@MWCNTs, and HFIP-α-Fe_2_O_3_@MWCNTs are shown in Figure 4b. Compared with the FT-IR pattern of the original MWCNTs, the MWCNTs oxidized by mixed acid show the strong C=O stretching vibrational peak and C-O vibrational peak of carboxyl group appeared at 1720 cm^−1^ and 1192 cm^−1^, respectively, and a broad peak around 3700 cm^−1^, which prove that the carboxyl groups (-COOH) on the surface of MWCNTs have significantly increased after acid treatment, this is conducive to the functionalization of HFIP groups. In the α-Fe_2_O_3_ spectrum, the stretching vibrations of the Fe-O bond are responsible for two strong and distinctive absorption bands at 466 and 560 cm^−1^. These findings affirm the successful synthesis of α-Fe_2_O_3_. Remarkably, the observation of the carbonyl stretch of the carboxylic acid group (C=O) at 1693 cm^−1^, C=C stretching vibration bands of the carbon skeleton at 1624 cm^−1^, the C-N amide carbonyl stretching vibration at 1572 cm^−1^, the C-F bending vibration band of -CF_3_ at 1438 cm^−1^, and the Si-O-Si vibrational peaks at 1086, 889, and 800cm^−1^ in the spectra of the final HFIP-α-Fe_2_O_3_@MWCNTs, show the successful functionalization of HFIP groups on α-Fe_2_O_3_@MWCNT composites [42,43,48]. The broad bands of 3845–3327 cm^−1^ are -OH bond stretching vibrations produced by the adsorption of water molecules by the material. The intensity of these peaks has enhanced after the HFIP-functionalized on α-Fe_2_O_3_@MWCNTs, confirming the successful synthesis of HFIP-α-Fe_2_O_3_@MWCNTs. After grafting HFIP, some of the adsorption bands disappeared while others, such as C=O, decreased in intensity. This phenomenon may be due to the depletion of -COOH groups in the carboxy-terminated MWCNTs through the formation of amide bonds [49].

**Figure 5:**
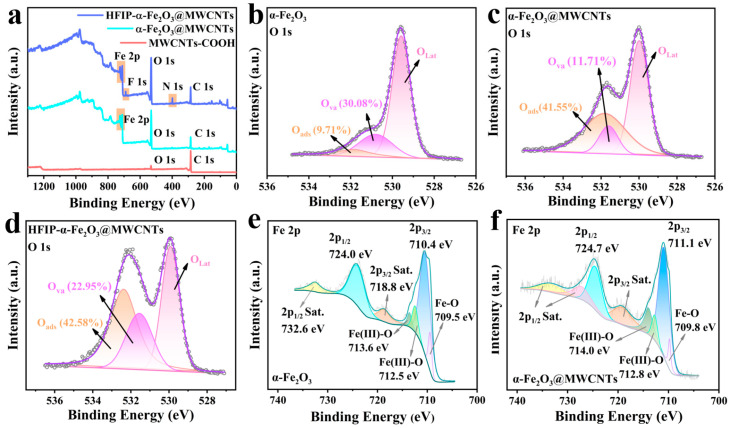
(**a**) Survey XPS spectra of different samples; high-resolution XPS spectrum of O 1s of α-Fe_2_O_3_ (**b**), α-Fe_2_O_3_@MWCNTs (**c**), and HFIP-α-Fe_2_O_3_@MWCNT composites (**d**); high-resolution XPS spectrum of Fe 2p of α-Fe_2_O_3_ (**e**) and α-Fe_2_O_3_@MWCNTs (**f**).

Furthermore, X-ray photoelectron spectroscopy (XPS) was applied to analyze the chemical structure and morphology of the sample materials. The XPS spectra of the three materials are shown in Figure 5a. As expected, MWCNT-COOH consists of the elements C and O, which exist in their chemical states of O 1s and C 1s, respectively, supporting their graphite-like structure. The element Fe in the composites exists in the state of Fe 2p. After functionalization with HFIP, peaks of N1s and F1s appeared in the spectra of HFIP-α-Fe_2_O_3_@MWCNTs, which conformed to the HFIP groups. The XPS spectra of C 1s peaks of the three samples are shown in Figure S2. The MWCNTs in α-Fe_2_O_3_@MWCNTs and HFIP-α-Fe_2_O_3_@MWCNTs were oxidized by the mixed acid, and the change in C atom binding resulted in the shift of the C-C peak. Detailed analysis of the C 1s spectra of HFIP-α-Fe_2_O_3_@MWCNTs reveals two new peaks in addition to the main peak at 285.05 eV, both of which can be attributed to the carboxyl groups introduced by the oxidation of the mixed acids. The peak of the Fe 2p in the composite is presented in Figure 5e. The peaks located at 724.0 eV and 710.4 eV can be associated with the characteristic signals of Fe 2p_1/2_ and 2p_3/2_, respectively. The two broad peaks at 732.6 eV and 718.8 eV are satellite peaks of Fe 2p_1/2_ and Fe 2p_3/2_, respectively [50,51]. The peak of Fe 2p_3/2_ could be divided into three peaks at binding energies of 713.6, 712.5, and 709.5 eV, respectively. These three peaks indicated the presence of an Fe-O bond [52]. After the functionalization of α-Fe_2_O_3_@MWCNTs, the peaks of Fe remained almost unchanged (shown in Figure 5f), indicating that functionalization did not affect the composition of the material. As is known, the oxygen adsorption ability is extremely crucial for surface resistive gas-sensing materials [53]. The high-resolution XPS O 1s peak spectra of the three materials (Figure 5b–d) show that they can all be separated into three peaks, corresponding to three different states of oxygen. The oxygen peak that is near 530.1 eV is attributed to lattice oxygen (O_Lat_), the peak near 530.8 eV is assigned to vacancy oxygen (O_Va_), and the peak near 532.1 eV is attributed to chemisorbed oxygen or free oxygen (O_abs_). By calculating the intensity of each peak in the O 1s spectrum, the percentage of different oxygen content in the sample can be estimated. Their relative content can be found in Table S1 in Supplementary Materials. The relative contents of O_Va_ of single α-Fe_2_O_3_, α-Fe_2_O_3_@MWCNTs composite, and HFIP-α-Fe_2_O_3_@MWCNTs are 30.08%, 11.71%, and 22.95%, respectively. In contrast, the relative contents of O_abs_ in the samples are 9.71%, 41.55%, and 42.58%, respectively. Compared with single α-Fe_2_O_3_, the relative content of O_Lat_ in α-Fe_2_O_3_@MWCNTs and HFIP-α-Fe_2_O_3_@MWCNT composites decreased significantly, and the remaining oxygen content almost doubled. In the α-Fe_2_O_3_@MWCNT composite, the heterojunctions formed by MWCNTs and α-Fe_2_O_3_ produced a substantial quantity of lattice mismatches, which introduced a great deal of vacancy oxygen (O_Va_) and chemisorbed oxygen (O_abs_). The composite was treated with acid before the grafting of HFIP, so the oxygen content was further increased [54]. The increase in the amount of oxygen O_Va_ and O_abs_ makes the sensitive materials absorb more oxygen involved in the gas-sensing reaction, thereby increasing their response to the corresponding gas.

**Figure 6:**
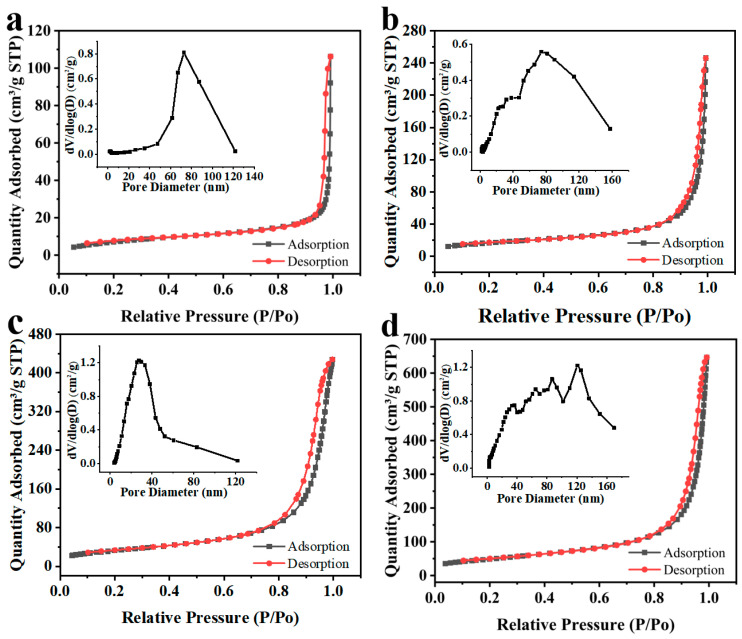
Nitrogen adsorption–desorption isotherms and Barrett–Joyner–Halenda (BJH) pore size–volume distribution (inset) of MWCNTs (**a**), α-Fe_2_O_3_ (**b**), α-Fe_2_O_3_@MWCNTs (**c**), and HFIP-α-Fe_2_O_3_@MWCNTs (**d**).

In order to further examine the pore structure, specific surface area and pore size distribution of the materials, we also carried out BET measurement, and the N_2_ adsorption–desorption isotherms and pore size distributions of the four materials are displayed in Figure 6. The prepared nanoparticles presented a type-II isotherm curve with an H3 hysteresis loop based on the IUPAC classification, reflecting the presence of macropore in the sample. Furthermore, the stacking of the samples led to the formation of pores. Compared with the original MWCNTs (27.829 m^2^/g) and α-Fe_2_O_3_ (59.711 m^2^/g), the composite α-Fe_2_O_3_@MWCNTs and HFIP-α-Fe_2_O_3_@MWCNTs have a larger surface area of 118.442 m^2^/g and 124.796 m^2^/g, respectively, due to the composite structure and porous structure. The pore volumes of MWCNTs, α-Fe_2_O_3_, α-Fe_2_O_3_@MWCNTs, and HFIP-α-Fe_2_O_3_@MWCNTs are 0.165, 0.380, 0.690, and 1.018 cm^3^/g, respectively. The pore sizes of α-Fe_2_O_3_@MWCNTs (21.854 nm) and HFIP-α-Fe_2_O_3_@MWCNT (21.668 nm) composites are smaller than that of the original MWCNTs (20.471 nm) and α-Fe_2_O_3_ (23.975 nm). The results show that α-Fe_2_O_3_@MWCNT composites have higher porosity and high specific superficial area, which are favorable for effective gas adsorption and desorption.

## 4. Gas Sensing Properties

**Figure 7:**
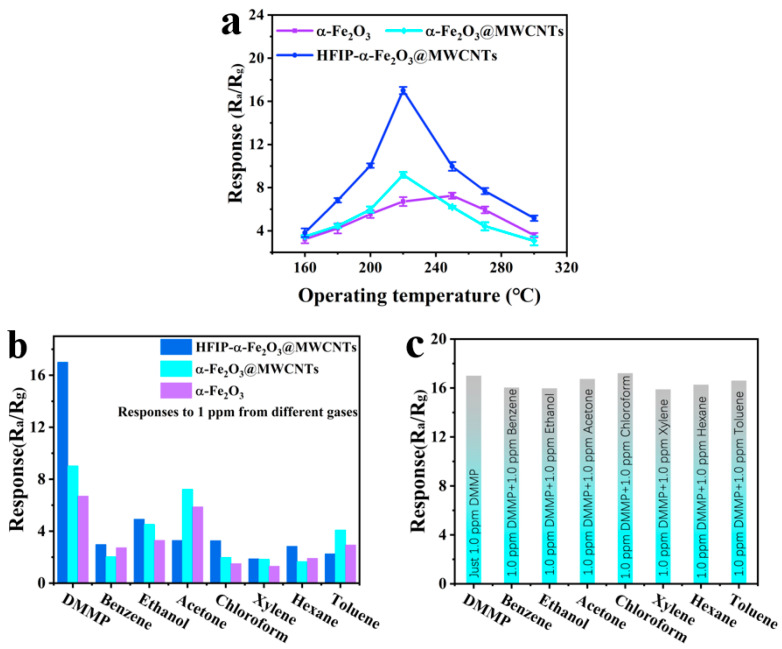
(**a**) The responses of α-Fe_2_O_3_, α-Fe_2_O_3_@MWCNTs, and HFIP-α-Fe_2_O_3_@MWCNTs to 1 ppm DMMP versus the test temperature; (**b**) the responses of α-Fe_2_O_3_, α-Fe_2_O_3_@MWCNTs, and HFIP-α-Fe_2_O_3_@MWCNTs toward various gases; (**c**) the responses of HFIP-α-Fe_2_O_3_@MWCNTs toward DMMP in the presence of other interfering gases.

The application of gas sensors requires thorough consideration of a number of parameters, the first of which is the operating temperature. We investigated the relationship between the operating temperatures and the responses to 100 ppm DMMP for the sensors based on the α-Fe_2_O_3_, α-Fe_2_O_3_@MWCNTs, and HFIP-α-Fe_2_O_3_@MWCNTs within the temperature range of 160–300 °C. The results are presented in Figure 7a, which demonstrated that the response of all sensors demonstrated a trend of initially rising and falling subsequently. At low operating temperatures, the gas-sensitive material demonstrates low surface activity. In turn, the activation energy present in the target gas molecules is insufficient to cause adsorption on the sensing material, leading to low sensitivity. As the operating temperature increases, the surface activity and the amount of chemisorbed oxygen of the sensitive material increase, and a higher response value is obtained. However, when the temperature is too high, the adsorption rate and desorption rate of chemical-adsorbed oxygen on the surface of the sensitive material decrease, which reduces the amount of chemical-adsorbed oxygen on the surface of the material, reducing the sensor response. According to the experimental results, the optimal operating temperatures of α-Fe_2_O_3_, α-Fe_2_O_3_@MWCNTs, and HFIP-α-Fe_2_O_3_@MWCNTs are 250 °C, 220 °C, and 220 °C, and the response values are 7.2, 9.0, and 15.7, respectively. The MWCNTs in the composite not only reduce the operating temperature but also provide pathways for carriers in the reaction and reduce the response time. The gas-sensitivity of HFIP-α-Fe_2_O_3_@MWCNTs is 1.74 times greater than that of α-Fe_2_O_3_@MWCNTs and 2.18 times higher than α-Fe_2_O_3_, clearly indicating that the formation of heterojunction of α-Fe_2_O_3_@MWCNT composites as well as the functionalization of HFIP improved the sensitivity to DMMP.

Selectivity is a key parameter that affects the application prospects of gas sensors. To elucidate the selectivity of the sensitive materials toward DMMP, the responses of three types of sensors to 1 ppm of multiple volatile organic compounds (VOC) gases are shown in Figure 7b, including benzene, ethanol, chloroform, xylene, acetone, hexane, and toluene. It has been observed that the performance of the HFIP-α-Fe_2_O_3_@MWCNT sensor toward DMMP is much higher than that of the other analytes and other sensors. These observations indicate that the specific selectivity of HFIP-α-Fe_2_O_3_@MWCNTs for DMMP may be attributed to the hydrogen bonding interactions between the HFIP groups and the DMMP molecule. Furthermore, the similarity between the responses to DMMP in the systems with other gas molecules and only DMMP in the atmosphere is evident. The findings depicted in Figure 7c provide additional evidence of the DMMP selectivity and confirm that the potential presence of interfering gases in the operating environment does not impact the detection of DMMP molecules, making the sensor more resistant to interference during practical use.

**Figure 8:**
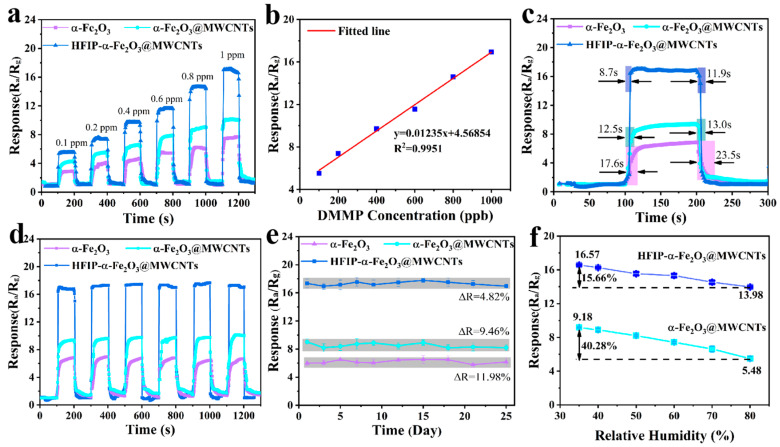
(**a**) The dynamic response curves of the α-Fe_2_O_3_, α-Fe_2_O_3_@MWCNT, and HFIP-α-Fe_2_O_3_@MWCNT sensors in the concentration range 0.1–1 ppm DMMP at their working temperature; (**b**) the responsivity of the HFIP-α-Fe_2_O_3_@MWCNT sensor versus DMMP concentration and the linear fitting results; (**c**) response–recovery times with 1 ppm DMMP concentrations; (**d**) multiple-cycle test curves of sensors at their working temperature with 1 ppm DMMP; (**e**) long-term stability of sensors at their working temperature with 1 ppm DMMP; (**f**) responses of the sensors at different values of RH.

Within 0.1–1.0 ppm, the dynamic responses of the sensors toward DMMP concentration (Figure 8a) show that all the sensors, especially the HFIP-α-Fe_2_O_3_@MWCNT sensor, have an outstanding correspondence, with the increase in sensor increasing almost linearly with DMMP concentration. The HFIP-α-Fe_2_O_3_@MWCNT sensor showed the highest response, almost twice that of the α-Fe_2_O_3_@MWCNT sensor. At a 1 ppm DMMP concentration, the response value of the HFIP-α-Fe_2_O_3_@MWCNT sensor is 16.8 (R_a_/R_g_), while the α-Fe_2_O_3_@MWCNT sensor is 7.4 (R_a_/R_g_), the response values of the α-Fe_2_O_3_ sensor and the MWCNT sensor are 6.5 (R_a_/R_g_) and 1.2 (R_g_/R_a_), respectively. The excellent detection performance of HFIP-α-Fe_2_O_3_@MWCNT for DMMP may be ascribed to the grafted HFIP groups of the sensitive material and the already existing carboxyl groups on the surface that can both form H-bonds with the receptor group (-OCH_3_) of DMMP. Compared with the original α-Fe_2_O_3_ sensor and MWCNT sensor, the α-Fe_2_O_3_@MWCNT sensor has a better gas response because of the larger specific superficial area of the composite, which offers more active sites for the chemical reaction. The results of the MWCNT sensor performance tests are shown in Figure S4. In order to calculate the limit of detection (LOD) and limit of quantitation (LOQ) of the HFIP-α-Fe_2_O_3_@MWCNT sensor, we performed a linear fit between the response value of HFIP-α-Fe_2_O_3_@MWCNT and the DMMP concentration in the range of 0.1–1.0 ppm (Figure 8b). The fitting line can be expressed as Y = 0.01235X + 4.56854, where the fitting coefficient R^2^ was 0.9951, indicating the high linearity. It was calculated that the LOD and LOQ of the HFIP-α-Fe_2_O_3_@MWCNT sensor are 63.92 ppb and 213.07 ppb, respectively [55].

Response time and recovery time reflect the speed of response and recovery of the gas sensor to the detected gas and are important factors in examining the performance of the sensor. They can be calculated based on the results of dynamic testing, presented in Figure 8c. The results demonstrated that the HFIP-α-Fe_2_O_3_@MWCNT sensor exhibited rapid response (8.7 s) and recovery (11.9 s) times compared to the other sensors. The composite formation and HFIP functionalization provide more oxygen vacancies for the sensitive materials, promote carrier conversion, and accelerate the surface redox reaction, thus improving the response time of the materials.

In practical applications, the long-term stability of gas sensors is a crucial indicator of sensor performance, hence the sensors were subjected to several cycles of testing under normal operating conditions to check their stability. Six detection cycles at the operating temperature were carried out at 1 ppm DMMP (indicated in Figure 8d). The findings show that the sensing performances of all sensors after several cycles are almost identical, with similar curve shapes, response–recovery behaviors, and sensitivity values. As shown in Figure 8e, all the sensors were tested for a long-term period of 25 days at a concentration of 1 ppm DMMP. The response of the HFIP-α-Fe_2_O_3_@MWCNT sensors showed a fluctuation of about 4.82%, which is almost negligible, further confirming its good reversibility and long-term stability. In comparison, the α-Fe_2_O_3_@MWCNT and α-Fe_2_O_3_ sensors fell by around 9.46% and 11.98%, respectively. Compared to other sensors, MWCNTs sensors are stable, although they do not have high response values (shown in Figure S5). These results indicate that the HFIP-α-Fe_2_O_3_@MWCNT sensing material can be utilized as a long-life and highly efficient DMMP sensor with practical applications.

Considering the practical application of DMMP sensors, the gas sensors may work under different weather conditions, and humidity may affect their performance. Therefore, the sensing performance of the α-Fe_2_O_3_@MWCNT and HFIP-α-Fe_2_O_3_@MWCNT samples were monitored under different relative humidity (RH) environments, and the results are shown in Figure 8f. It is clear that the responses of the sensors both decreased with the rise of humidity. At 80% RH, there is a significant decrease in the response of the sensor. The responses of HFIP-α-Fe_2_O_3_@MWCNTs and α-Fe_2_O_3_@MWCNTs are 13.98 and 5.48, respectively, which decreased by about 15.66% and 40.28%. These results indicate that the HFIP-α-Fe_2_O_3_@MWCNTs still have high response stability over a wide range of RH and function perfectly under both dry and humid conditions.

The performances of recent works employing graphene and carbon nanotubes hybrids for DMMP detection are compared with HFIP-α-Fe_2_O_3_@MWCNTs, shown in Table 1. It can be seen that HFIP-α-Fe_2_O_3_@MWCNTs have a higher response and a faster response recovery time, demonstrating the competitional performance of this material for chemical warfare agent (CWA) sensing.

## 5. Gas Sensing Mechanism

**Figure 9:**
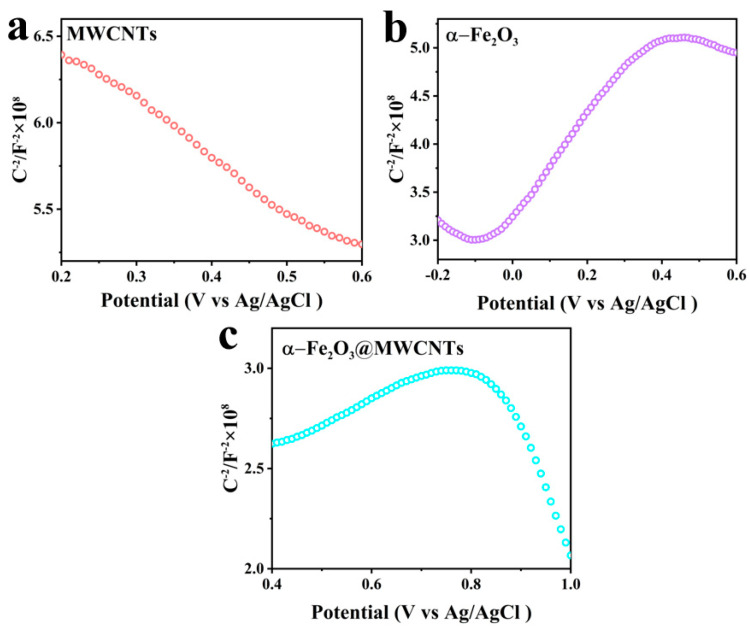
Mott–Schottky plots of MWCNTs (**a**), α-Fe_2_O_3_ (**b**), and α-Fe_2_O_3_@MWCNTs (**c**).

Semiconductor gas sensors are made by using the redox reaction of gases on the surface of semiconductors to cause changes to the resistance value of the sensitive element. Under certain conditions (e.g., temperature), the gas to be measured reaches the surface of the material and reacts chemically with the oxygen adsorbed on the surface accompanied by charge transfer, which further causes a change in the semiconductor resistance, which can be realized through the measurement of the change in semiconductor resistance to the detection of gas [28,60]. P-N junctions are at the heart of many semiconductor devices. MWCNTs is a p-type semiconductor while α-Fe_2_O_3_ is an n-type, so heterojunctions can easily form at the interface of the two materials. To verify the conjecture, Mott–Schottky curves were tested for MWCNTs, α-Fe_2_O_3,_ and α-Fe_2_O_3_@MWCNTs, as shown in Figure 9. A tangent line is made to the longest straight part of the Mott–Schottky curve, and the slope of the tangent line of α-Fe_2_O_3_ is positive, indicating that the semiconductor is an n-type semiconductor; the slope of the tangent line of MWCNTs is negative, indicating that the material is a p-type semiconductor. The α-Fe_2_O_3_@MWCNT composite material has the curve of an inverted V, indicating that there exists an n-p heterojunction in this material [61]. The n-p heterojunction formed between them is the main factor that enhances the response of the composite.

**Figure 10:**
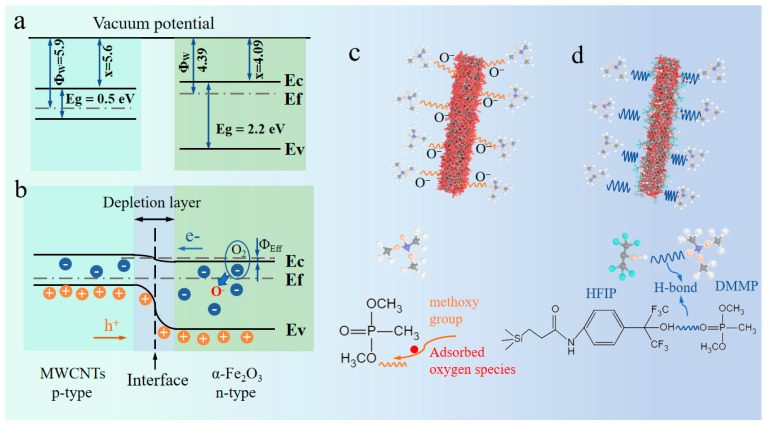
(**a**) Energy band diagram of α-Fe_2_O_3_ and MWCNTs; (**b**) energy band diagram of the α-Fe_2_O_3_@MWCNT composite; schematic diagram of the DMMP sensing mechanism of sensors based on αFe_2_O_3_@MWCNT composites (**c**), and hybrid HFIP-α-Fe_2_O_3_@MWCNTs (**d**). The abbreviation Ec is the conduction band, Ev is the valence band, Ef is the Fermi level, Eg is the energy bandgap, Φw is the work function, and χ is the electron affinity.

According to previous literature reports, the calculated band gap and figure of merit of p-type MWCNTs are about 5.6 and 0.5 eV [62], respectively, and the electron affinity and band gap of n-type α-Fe_2_O_3_ are 4.78 and 2.2 eV [63], respectively. The energy band diagrams of the two materials are shown in Figure 10a. The electronic structure and energy band shifts of materials are revealed by the VB-XPS test (Figure S5). The valence band positions with respect to the vacuum (E_VB-vac_) are obtained from the VB-XPS spectra, and the valence band (VB) positions of α-Fe_2_O_3_, α-Fe_2_O_3_@MWCNTs, and HFIP-α-Fe_2_O_3_@MWCNTs, with respect to the standard hydrogen electrode (E_VB-NHE_), are calculated to be 2.42, 2.53, and 2.56 eV, respectively (calculation shown in Supplementary Materials), suggesting that that grafting HFIP groups introduce electron holes in α-Fe_2_O_3_@MWCNTs, which broaden the VB width. After α-Fe_2_O_3_ modification of MWCNTs, an n-p heterojunction was formed at the interface of the two materials. The energy band diagram of α-Fe_2_O_3_@MWCNT composites is shown in Figure 10b. In the air atmosphere, free oxygen molecules (O_2_) are adsorbed onto the surface of the sensitive material. Due to the high electron affinity of oxygen, electrons are transferred from the gas-sensitive material to the oxygen molecules, forming oxygen ions. The generation of oxygen ions is related to the temperature of the gas-sensitive material. At low temperatures, the oxygen ions will be in the ($O_{2}^{-}$) chemisorbed state, whereas at high temperatures, they will be in the O^−^ or O^2^ states [46]. Consequently, electrons transfer from the surface of the gas-sensitive material to the oxygen molecule, thereby causing the formation of an electron depletion layer on the surface of the n-type material (α-Fe_2_O_3_). Similarly, a hole depletion layer is formed on the p-type crystals (MWCNTs), leading to a decrease in the carrier concentration inside the gas-sensitive material, and resulting in the formation of a broad depletion layer on the surface of the composite material and an increase the resistance in the sensors circuit [64], as indicated in Figure 10a. In the heterojunction regions, many holes (h^+^) will transfer from MWCNTs to α-Fe_2_O_3_ which naturally reduce the height of the potential barrier between the crystals below that of pristine α-Fe_2_O_3_ and MWCNTs, increasing the sensitivity [65]. The energy band gap diagram (Figure 10b) demonstrates that the high barrier (Φ_eff_) on the crystals’ surface prevents the charge of the charge carriers. Upon exposing the sensors to the DMMP environment, the DMMP molecules will react with adsorbed oxygen species, releasing the captured electrons back into the conduction band. The enriching of the conduction band with electrons causes an increase in the charge carrier in the sensing materials, forming an electron accumulation region on the surface of sensing materials [66]. The reaction between the DMMP molecules and adsorbed oxygen species ($O_{2}^{-}$) on the MWCNTs (p-types) and α-Fe_2_O_3_ (n-type) crystals can be described by Equations (1) and (2) [67].

C_3_H_9_O_3_P + 5O_2_ + 10h^+^ → H_3_PO_4_ + 3CO_2_ + 3H_2_O (1)

C_3_H_9_O_3_P + 5O_2_ → H_3_PO_4_ + 3CO_2_ + 3H_2_O + 10e^−^ (2)

After the reaction, the decrease in the depletion layer thickness and the energy barrier height (Φ_eff_) leads to a decrease in the resistance in the sensor’s circuit until the conduction band (CB) of the sensing materials is saturated with free electrons [68]. After the performance has stabilized, the sensor is transferred to an air environment where the sensitive material re-adsorbs oxygen ions, which makes the resistance return to its initial value. These processes will be performed during each test cycle. The above findings indicate that the crystal structure and chemical composition of α-Fe_2_O_3_@MWCNTs hierarchical nanoheterostructure provide a large number of active sites and rapid channels for carriers. The formation of the n-p heterojunction reduces the height of the inter-crystalline energy barriers, which reduces the sensing temperature and makes the sensing speed faster.

Moreover, the layered structure of the α-Fe_2_O_3_@MWCNT composite in this experiment is also one of the factors contributing to the enhanced gas-sensitive properties. The structural characteristics of the α-Fe_2_O_3_@MWCNT composite make it have a large specific superficial area and better permeability. According to the SEM and TEM characterization of the material (Figure 3) and the BET (Figure 6), the polycrystalline α-Fe_2_O_3_ nanorods are loosely stacked on the surface of the MWCNTs skeleton to form a multilevel layered structure with a large specific superficial area, and compared with that of the individual α-Fe_2_O_3_ nanorods (58.097 m^2^/g), the specific superficial area of the α-Fe_2_O_3_@MWCNTs composites was 114.345 m^2^/g. These results mean that the material can offer further active sites for the reaction, which come into contact with more oxygen molecules and absorb and ionize more oxygen molecules, thus increasing the utilization of sensitive materials [69]. The excellent electrical properties of MWCNTs provide a direct pathway for charge carriers, further reducing the response—recovery time. Therefore, compared with pure α-Fe_2_O_3_ and bare MWCNTs sensors, α-Fe_2_O_3_@MWCNTs composite sensor demonstrates stronger response and fast response/recovery characteristics.

When the sensor is exposed to DMMP gas, the DMMP molecules will bind to the oxygen adsorbed on the surface of α-Fe_2_O_3_@MWCNTs composites through methoxy (O-CH_3_). Since DMMP is an outstanding electron donor, the electrons will be transferred to the sensing material, thus reducing the conductivity (shown in Figure 10c) [70]. Because different molecules can all bind to the metal oxides through methoxy bonds, this interaction cannot achieve DMMP-specific detection. According to Lewis’s acid-base theory, DMMP is a hydrogen-bonded alkaline gas, while the HFIP group has enhanced selective adsorption capacity for DMMP as a hydrogen-bonded acidic organic matter. After the composite is functionalized with HFIP, HFIP (-O-H) can form hydrogen bonds with DMMP (-P=O). In addition, -COOH on the surface of the composite can also interact with DMMP to form hydrogen bonds [57]. Therefore, the double hydrogen bonding interactions with DMMP of HFIP-α-Fe_2_O_3_@MWCNTs showed higher adsorption and specific selectivity.

## 6. Conclusions

In this paper, we report a highly selective and excellently performant DMMP sensor based on HFIP-α-Fe_2_O_3_@MWCNTs. By assembling α-Fe_2_O_3_ nanorods on the skeleton of carbon nanotubes, a novel α-Fe_2_O_3_@MWCNTs layered heterostructure is prepared. The characterization results show that the prepared material has high porosity and specific surface area. On this basis, it was functionalized by HFIP and its selectivity to DMMP gas was improved. At the optimum operating temperature, the HFIP-α-Fe_2_O_3_@MWCNT composite sensor responds to 1 ppm of DMMP with a ratio of 16.8 (R_a_/R_g_), while that of the unfunctionalized α-Fe_2_O_3_@MWCNT composite is 9.1 (R_a_/R_g_). The response values of the α-Fe_2_O_3_ and MWCNTs are 6.5 (R_a_/R_g_) and 1.2 (R_g_/R_a_), respectively. Compared with the original MWCNTs and α-Fe_2_O_3_ sensors, the composite α-Fe_2_O_3_@MWCNT sensor has a faster response/recovery time and better performance due to the heterojunction formation between the two materials. The response of the functionalized sensing material to DMMP is further improved due to the hydrogen bonding with HFIP by DMMP. The improved performance of the HFIP-α-Fe_2_O_3_@MWCNT composite sensor is due to the formation of heterojunction, the enhancement of chemical oxygen absorption capacity, and the increase in the specific superficial area of the α-Fe_2_O_3_@MWCNTs’ hierarchical nanoheterostructure material. In summary, the novel layered DMMP sensing material (HFIP-α-Fe_2_O_3_@MWCNTs) is expected to promote the development and application of organic-inorganic hybrid materials as high-performance gas sensing materials.

## Figures and Tables

**Table 1:** Performance of the HFIP-α-Fe_2_O_3_@MWCNT sensor in comparison with DMMP sensors based on graphene and carbon nanotube hybrids.

Sensor Types	Sensing Materials	Operation Temperature	DMMP Concentration	Response Value	Response/Recovery Time (s)	References
SAW	NGO@MnO_2_/PPy	RT	25 ppm	98 Hz	120/197 (75 ppm)	[56]
QCM	MnO_2_@NGO/PPy	RT	50 ppm	87 Hz	101/123	[57]
Resistance	PPy-rGO	RT	100 ppm	12.9%	43/75	[4]
Resistance	β-MnO_2_@CNF	RT	100 ppb	22.7%	−/−	[58]
Resistance	CoPc-HFIP-GQD	RT	20 ppm	8.4%	600/640	[59]
Resistance	PANI nanofiber/graphene	RT	3 ppb	1.9%	2/35	[7]
Resistance	rGO/WO_3_-HFIP	150 °C	10 ppm	17.6	9.4/12.6	[10]
Resistance	HFIP-α-Fe_2_O_3_@MWCNTs	225 °C	1 ppm	16.8	8.7/11.9	This work

## Data Availability

The data that support the findings of this study are available from the corresponding author upon reasonable request.

## Supplementary Materials

The following supporting information can be downloaded at: https://www.mdpi.com/article/10.3390/nano14030305/s1, Figure S1. SEM images of the ceramic tube covered by sensing material in different magnifications confirm the full covering of tubes by sensing material. Figure S2. (a) High-resolution XPS spectrum of C 1s of MWCNTs, α-Fe_2_O_3_@MWCNTs and HFIP-α-Fe_2_O_3_@MWCNTs composites; (b) High-resolution XPS spectrum of C 1s of HFIP-α-Fe_2_O_3_@MWCNTs. Table S1. The atomic percentage for O elements of prepared samples was obtained from XPS analysis. Figure S3. Multiple-cycle test curves of MWCNTs sensor at RT with 1 ppm DMMP. Figure S4. Long-term stability of MWCNTs sensors at RT with 1 ppm DMMP. Figure S5. VB-XPS spectra of α-Fe_2_O_3_, α-Fe_2_O_3_@MWCNTs and HFIP-α-Fe_2_O_3_@MWCNTs. Refs. [71,72] are cited in the supplementary materials.
