# Supplementary material for: Hierarchical Nanoheterostructure of HFIP-Grafted α-Fe2O3@Multiwall Carbon Nanotubes as High-Performance Chemiresistive Sensors for Nerve Agents
Source: Nanomaterials (Basel). 2024 Feb 2;14(3):305. doi: 10.3390/nano14030305 (PMC10857011; doi:10.3390/nano14030305)
Supplement: Supplementary file 1 [file nanomaterials-14-00305-s001.zip › nanomaterials-2809116-supplementary.pdf]

Supplementary Materials

Hierarchical Nanoheterostructure of HFIP-Grafted α-Fe_2O_3@Multiwall Carbon Nanotubes as High-Performance Chemiresistive Sensors for Nerve Agents

Xuechun Wang, Jingyuan Liu *, Rumin Li, Jing Yu, Qi Liu, Jiahui Zhu, Peili Liu *

Key Laboratory of Superlight Material and Surface Technology, College of Materials Science and Chemical Engineering, Harbin Engineering University, Harbin 150001, China.

*Correspondence: liujingyuan1004@hrbeu.edu.cn (J.L.); liupeili@hrbeu.edu.cn (P.L.)

The supplementary materials include:

1. Section of the chemical reagents
2. Section of characterization equipments.
3. Section of gas sensing measurements.
4. (Figure S1) SEM images of the ceramic tube covered by sensing material in different magnifications confirm the full covering of tubes by sensing material.
5. (Figure S2) (a) High-resolution XPS spectrum of C 1s of MWCNTs, α-Fe_2O_3@MWCNTs and HFIP-α-Fe_2O_3@MWCNTs composites; (b) High-resolution XPS spectrum of C 1s of HFIP-α-Fe_2O_3@MWCNTs.
6. (Table S1) The atomic percentage for O elements of prepared samples was obtained from XPS analysis.
7. (Figure S3) Multiple-cycle test curves of MWCNTs sensor at RT with 1 ppm DMMP.
8. (Figure S4) Long-term stability of MWCNTs sensors at RT with 1 ppm DMMP.
9. (Figure S5) VB-XPS spectra of α-Fe_2O_3 ,α-Fe_2O_3@MWCNTs and HFIP-α Fe_2O_3@MWCNTs.

S1. Materials

All the applied chemical reagents are analytical grade and used directly without any further purification. Carboxylated multi-walled carbon nanotubes (MWCNTs) with a range of 8-15 nm in diameter and 0.5−2 μm in length were provided by XFNAN Co., Ltd. Concentrated sulphuric acid (H_2SO_4, 72%), aqua fortis (HNO_3, 68%), ethanol absolute (100 %) and N, N-Dimethylformamide (DMF, C_3H_7NO, MW:73.095)were purchased from Tianjin FuYu Fine Chemical Co., Ltd. Iron (III) chloride hexahydrate ($FeCl_3 \cdot 6H_2O$, MW=270.29) was purchased from Tianjin Hengxing Chemical Preparation Co., Ltd. Carboxyethylsilanetriol sodium salt (CES, $C_3H_7O_5Si.Na$, 25 wt% in water, MW:174.16) and hydrochloric acid (HCl, 99 wt%) were obtained from Shanghai Chemical Reagent Co. Ltd. Hydroxybenzotriazole (HOBt, $C_6H_5N_3O$) was purchased from J&K Scientific Chemical Reagent Co., Ltd. N-ethyl-N-(3-dimethylaminopropyl) carbodiimide hydrochloride (EDCl, $C_8H_{17}N_3 \cdot HCl$, 99wt%, MW:191.7) was purchased from Macklin. Triethylamine ($C_6H_{15}N$, 99wt%, MW: 101.19) was purchased from Tianli Chemical Reagent Co., Ltd. Hexafluoroacetone trihydrate (HFIP, $C_3F_6O \cdot 3H_2O$, MW:200.07) and Dimethyl methylphosphonate (DMMP, $C_3H_9O_3P$, 98 wt %, MW:124.08) were obtained from Aladdin.

S2. Characterization Equipments

The MWCNTs and prepared α-Fe_2O_3, α-Fe_2O_3@MWCNTs composites and HFIP-α-Fe_2O_3@MWCNTs nanostructure structure were characterized via powder X-ray diffraction (XRD, Rigaku TTR-III). Which operated at 40 kV/150 mA using high-intensity nickel-filtered copper Kα (K alpha) radiation (λ=0.15406 nm) in 2 Theta range 5-80°. Fourier Transform Infrared (FTIR) spectroscopy was recorded using Avatar 370 in the range of 500-4000 cm^{-1}; the samples were pressed with a standard KBr powder. The chemical composition was identified by X-ray photoelectron spectroscopy (XPS, Thermo ESCALAB 250Xi). The architecture and surface morphologies of the MWCNTs, after growth α-Fe_2O_3, and after grafting of the HFIP group were checked out via Scanning Electron Microscopy using Thermoscientific Apreo S SEM machine run at 10 kV accelerating voltage. The micro-nanostructure was observed via Transmission Electron Microscopy (TEM, Tecnai-G220S-Twin, FEITEM) at 120 kV accelerating voltage. Energy-Dispersive X-ray spectra (EDX) were obtained from the

Transmission Electron Microscopy. The specific surface area, porous volume, and porous distribution were calculated via single point Brunauer-Emmett-Teller (BET) equation and Barrett-Joyner-halenda (BJH) model from the Nitrogen adsorption-desorption isotherm curves using a Micromeritics ASAP 2010 M instrument at liquid nitrogen temperature. The gas sensing measurements were carried out using a commercial NMDOG multifunctional precision sensor analysis tester (manufactured in Changsha Dingcheng Scientific Instrument Co., Ltd, Hunan province, China) at room temperature and laboratory conditions.

S3. Measurement of the gas-sensing properties

The gas sensing measurements were carried out in the laboratory atmosphere at room temperature (in the range of 25-28 ºC) and relative humidity (in the range of 25-35 RH %). The sensing performance of the sensors was decided by taking an average of 3 samples from each sensing material. The sensors were firstly operated for 3 hours in the air ambient until the stable resistance behavior of the sensors was achieved. Then, a trace of the targeted gases in the liquid state was dropped inside a 100 mL three-necked flask by an assistant a microsyringe; the flask was already heated to the boiling point of the targeted gas. Then, after the complete evaporation of the gas sources, a valve between the flask and the 10 L closed bottle was opened, and the saturated gas was moved to the test bottle via a hot stream. Then, after running the sensors in the air for 100 s, the sensors's holder was quickly moved to the test bottle to measure the sensing response in the gas ambient. After 100 s, the sensors were taken out and kept running in the air for another 100 s to observe the recovery behavior. The test process was repeated in each test cycle under different gas concentrations and types. The concentration of the gases was calculated depending on the static liquid gas distribution method using the following mathematic equation (S1) [1, 2]:

$$C= \frac{0.082\times T\times \rho\times V_1}{M\times V_2}\times 1000 \quad (S1)$$

Since C (ppm) is the concentration of the tested gases. T(K) is the absolute temperature, ρ (g mL^{-1}) is the density of test gases in a liquid state. V_1 (μL) is the volume of injected liquid. M (g mol^{-1}) is the molecular weight of the tested gases in the liquid form. And V_2 (L) is the volume of the test bottle.

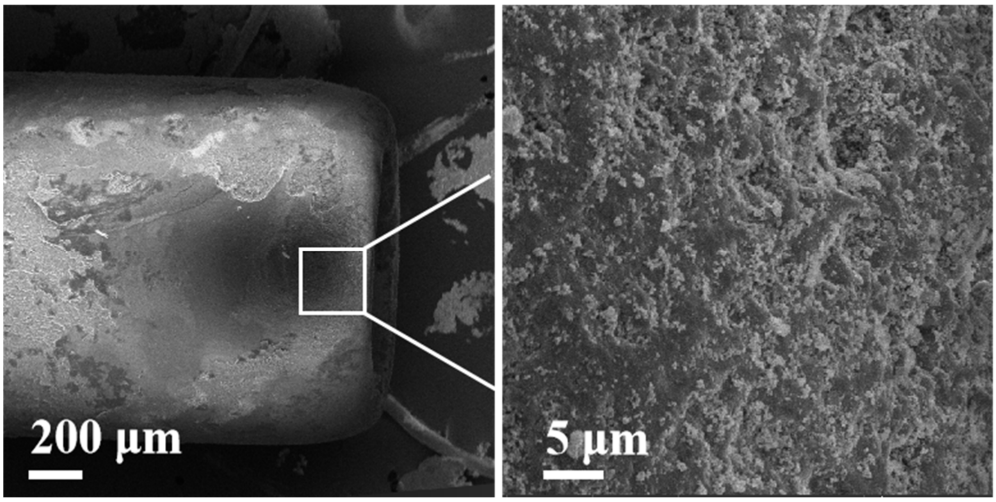

Figure S1. SEM images of the ceramic tube covered by sensing material in different magnifications confirm the full covering of tubes by sensing material.

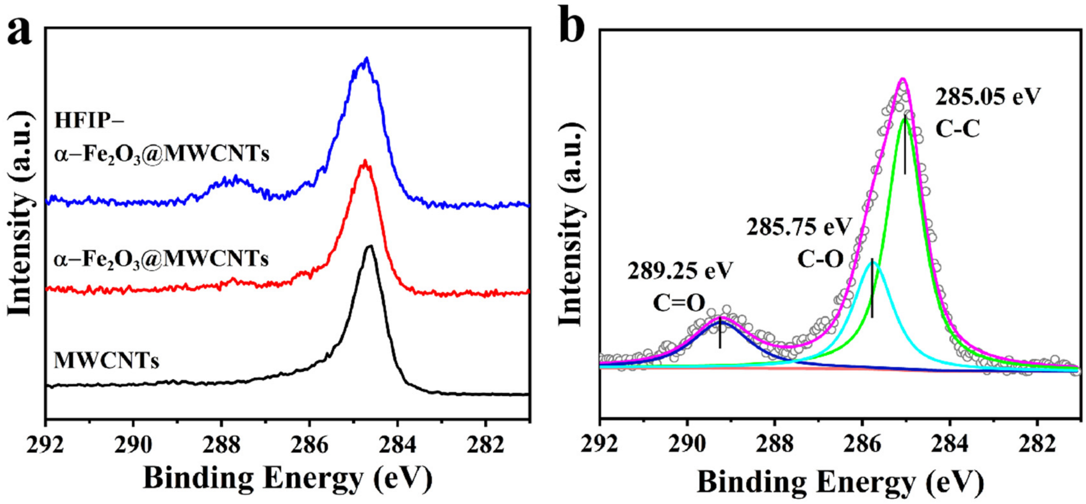

Figure S2. (a) High-resolution XPS spectrum of C 1s of MWCNTs, α-Fe_2O_3@MWCNTs and HFIP-α-Fe_2O_3@MWCNTs composites; (b) High-resolution XPS spectrum of C 1s of HFIP-α-Fe_2O_3@MWCNTs.

Table S1. The atomic percentage for O elements of prepared samples was obtained from XPS analysis.

Sample	BET surface Area (m^2g^{-1})	Pore volume (cm^3g^{-1})	Average pore size (nm)
MWCNTs	27.829	0.165	20.471
α-Fe_2O_3	59.711	0.380	23.975
α-Fe_2O_3@MWCNTs	118.422	0.690	21.854
HFIP-α-Fe_2O_3@MWCNTs	124.796	1.018	21.668

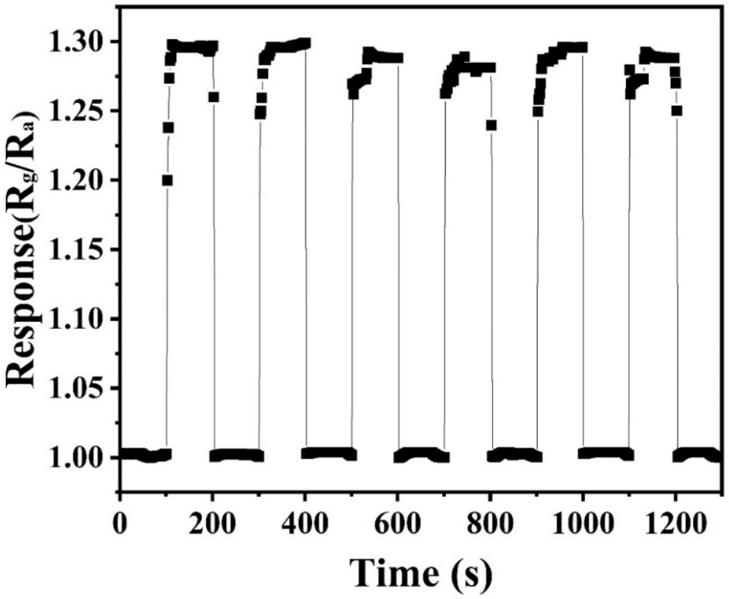

Figure S3. Multiple-cycle test curves of MWCNTs sensor at RT with 1 ppm DMMP.

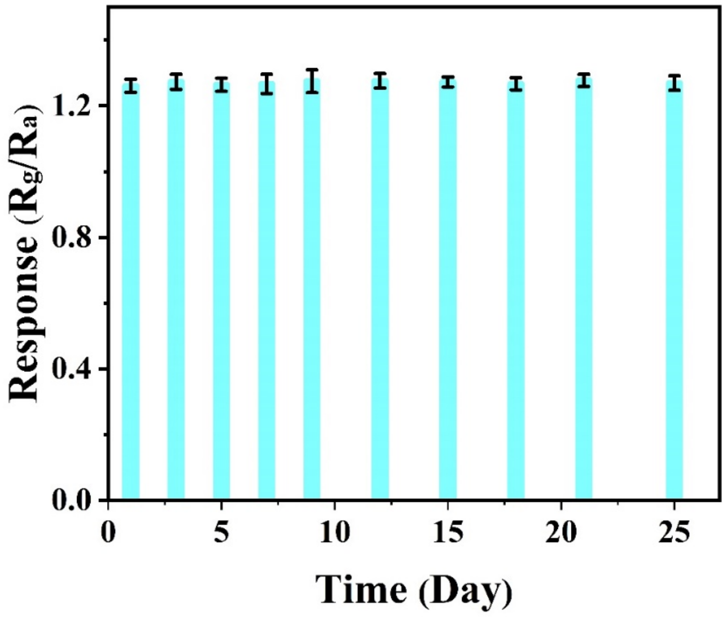

Figure S4. Long-term stability of MWCNTs sensors at RT with 1 ppm DMMP.

The VB obtained from the VB-XPS test is the valence band positions with respect to the vacuum ($E_{VB\text{-}vac}$), which can be converted to the valence band with respect to the standard hydrogen electrode ($E_{VB\text{-}NHE}$) using the following mathematic equation (S2):

$$E_{VB-NHE} = \varphi + E_{VB-vac} - 4.44 \quad (S2)$$

Since φ (eV) is the the instrumental work function.

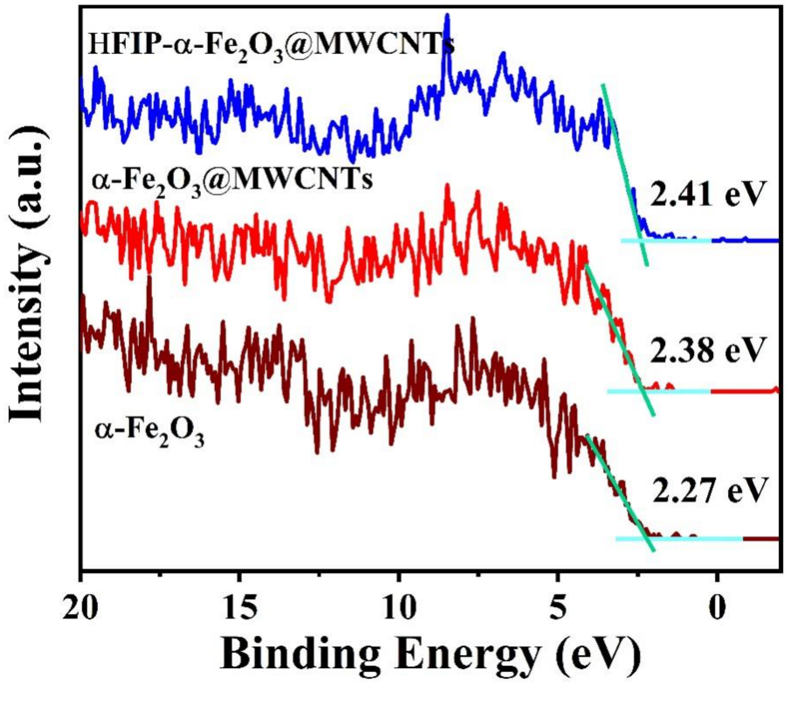

Figure S5. VB-XPS spectra of α-Fe_2O_3 ,α-Fe_2O_3@MWCNTs and HFIP-α-Fe_2O_3@MWCNTs.

References

[1] M. Imran, Y. Sabri, N. Motta, T. Tesfamichael, P. Sonar, M. Shafiei, Template based sintering of WO_3 nanoparticles into porous tungsten oxide nanofibers for acetone sensing applications, J. Mater. Chem. C, 7 (2019) 2961-2970.

[2] F. H. Saboor, T. Ueda, K. Kamada, T. Hyodo, Y. Mortazavi, A. A. Khodadadi, Y. Shimizu, Enhanced NO_2 gas sensing performance of bare and Pd-loaded SnO_2 thick film sensors under UV-light irradiation at room temperature, Sens. Actuators B-Chem., 223 (2016) 429-439.
